# Spatial and multi-omic profiling reveals genes and pathways associated with cytotoxic lymphocyte infiltration in malignant rhabdoid tumor

**DOI:** 10.21203/rs.3.rs-7303174/v1

**Published:** 2025-08-19

**Authors:** Marco Marra, Dan Jin, Joshua Dubland, Elizabeth Mullen, James Geller, Jonathan Bush

**Affiliations:** University of British Columbia; University of British Columbia; BC Children’s and Women’s Hospital; Dana-Farber Cancer Institute; Peckham Center for Cancer and Blood Disorders, Rady Children’s Hospital, San Diego, CA University of California San Diego, La Jolla, CA; University of British Columbia

**Keywords:** Malignant rhabdoid tumor, single-cell multiomics, spatial transcriptomics, tumor heterogeneity, tumor microenvironment, tumor antigens, tumor-associated macrophages

## Abstract

Malignant rhabdoid tumors (MRTs) are aggressive pediatric cancers with poor outcomes. MRTs exhibit low tumor mutational burden, yet recent studies reported immune cell infiltration. Here, we used spatial transcriptomics and multi-omic profiling to study immune cell infiltration in MRT samples. We observed a diverse repertoire of candidate tumor antigens (TAs), *IRF1* signaling activity and elevated expression of antigen processing and presentation genes, strongly associated with a “hot” tumor immune microenvironment (TIME). Upregulation of factors involved in skeletal muscle development was observed in some MRTs with higher CD8 + T cell infiltration and the ratio of M1/M2 macrophages was positively correlated with cytotoxic lymphocyte infiltration. We identified genes, such as *SPP1*, preferentially expressed in tumor-associated macrophages (TAMs) and noted the *MITF* regulatory network appeared active in the M2-like TAMs.

## Introduction

Malignant rhabdoid tumors (MRTs) are rare but aggressive pediatric cancers that frequently arise in the kidney (renal MRTs), soft tissues (extracranial, extrarenal MRTs, eMRTs), and central nervous system (atypical teratoid rhabdoid tumors, AT/RTs)^1–4^. Treatment options vary depending on the site and stage, but often include local control through surgery or radiation therapy, in conjunction with chemotherapy (often vincristine, actinomycin, and doxorubicin)^5^, with additional consideration for EZH2-inhibitors^6^. The 5-year overall survival rate is 27%^7^, indicating the need for an improved understanding of MRT vulnerabilities and therapies to target these.

Nearly all MRTs exhibit biallelic mutations in *SMARCB1* and, less commonly, biallelic mutations in *SMARCA4*, both of which encode core subunits of the switch/sucrose non-fermentable (SWI/SNF) chromatin-remodeling complex^8,9^. Similar to other pediatric malignancies, the somatic mutation rate in MRTs is low^10–12^. MRTs were thus initially believed to have minimal immune cell infiltration and be less likely to respond to immunotherapy^13^. However, studies using bulk and single-cell RNA-Seq have inferred the presence of tumor-infiltrating immune cells in MRT^14,15^. The levels of different infiltrating immune cells appeared correlated with different rhabdoid tumor molecular subgroups defined by DNA methylation and gene expression patterns^14–19^. Other studies investigated the mechanisms underlying the observed variation in immune cell infiltration levels. Leruste et al. reported that the immunogenicity of rhabdoid tumors, measured by the cytolytic activity (CYT) score^20^, is mediated by the re-expression of endogenous retroviruses and the activation of interferon signaling, arising from dysfunctional chromatin regulation as a result of SMARCB1 loss^14^, but previously we did not observe epigenetic derepression of retroviral elements^15^. Instead, we observed increased expression of immune-related genes, HOX genes, and mesoderm developmental regulators in MRT subgroups with higher levels of cytotoxic T cell infiltration^15^. We proposed that the increased T cell level observed in MRT “Group1 (ATRT-MYC-like)” was potentially attributable to the co-deletion of the immune modulator *MIF* along with *SMARCB1*^15^.

Macrophages are the most abundant immune cell population in rhabdoid tumors and are a negative prognostic factor for AT/RT patient survival^14–16^, raising the possibility that the composition of the tumor immune microenvironment (TIME) may play an important role in regulating the antitumor response. Tumor-associated macrophages (TAMs) participate in various aspects of cancer progression, metastasis, and resistance to treatment. TAMs are not a homogeneous entity but rather exist in a spectrum of activation states and have been broadly classified into the M1 and M2 two subgroups, which respectively have anti-tumor or pro-tumor activities^21,22^. Single-cell RNA sequencing (scRNA-seq) of fluorescence-activated cell sorted myeloid cells from one peripheral blood mononuclear cell (PBMC) sample and three AT/RT samples revealed heterogeneous TAM subpopulations^14^. However, AT/RTs only represent the subset of MRTs that occur in the central nervous system, and extracranial MRTs may possess different TIME compositions. Analysis of extracranial MRT TIME thus has the potential to provide new insights into resident immune cell populations.

To study tumor cells in relation to TIME, we profiled primary MRT samples using spatial transcriptomic and multi-omic approaches. We found that MRTs express several candidate tumor antigens (TAs) but TA expression did not correlate with inferred cytotoxic lymphocyte levels. We observed that the immune cell composition varied between intra-tumor regions, as well as across different tumors and that the inferred activity of the *IRF1* regulatory network and the expression of antigen processing and presentation genes were associated with cytotoxic lymphocyte infiltration. A subset of MRTs with high CD8 + T cell levels exhibited increased expression of a skeletal muscle development transcriptional program. M1/M2 macrophage ratios were positively correlated with cytotoxic lymphocyte infiltration, indicating the potential role of macrophages in modulating the immune response. We also identified genes and pathways preferentially associated with M2-like TAMs.

## Results

### Experimental Design

**Figure 1:**
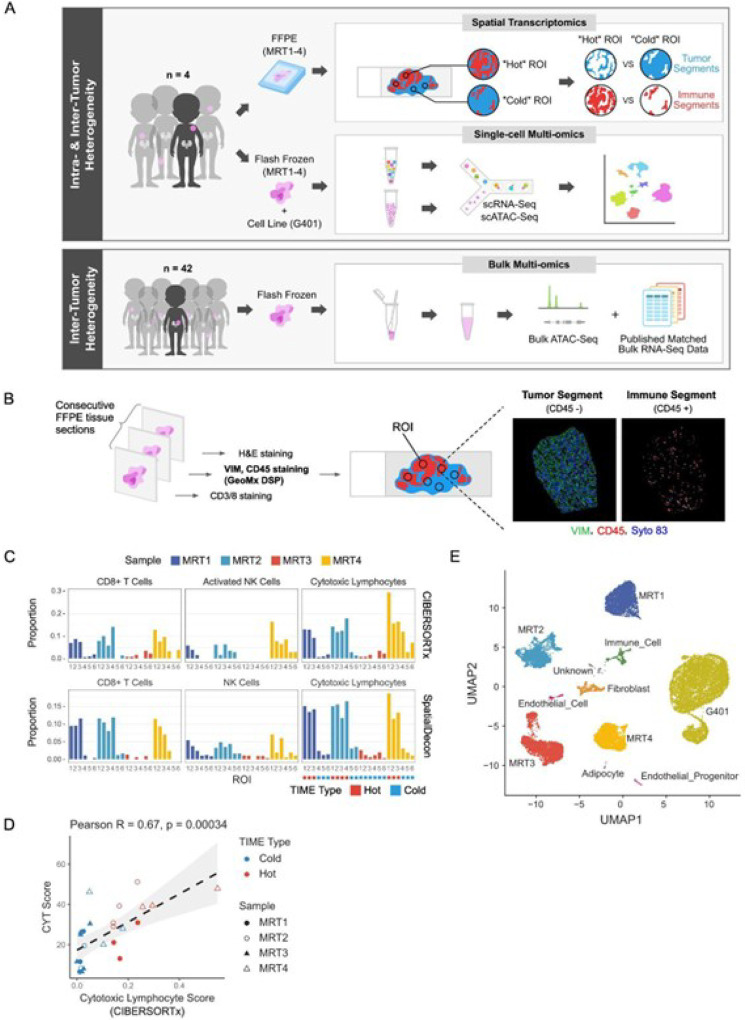
Experimental Design (See also Figures S1 and S2) A) Summary of tumor samples and methods used in this study. Four MRT samples were profiled using spatial transcriptomics and single-cell technologies. To compare and validate findings in a larger cohort, bulk RNA-seq data from 65 MRT samples^11^ were obtained, and bulk ATAC-seq data were generated for 42 samples from the same cohort. B) Overview of Bruker/NanoString GeoMX DSP spatial transcriptomics profiling strategy (Methods). C) Assignment of tumor immune microenvironment (TIME) types based on cell type deconvolution of the immune segments from the 24 regions of interest (ROIs) profiled with spatial transcriptomics. D) Scatterplot showing the correlation between CIBERSORTx-inferred cytotoxic lymphocyte infiltration and the cytolytic (CYT) score from spatial transcriptomic data. The grey band shows the 95% confidence interval. E) UMAP visualization and annotation of clusters from single-cell multi-omic data (Methods).

We profiled 4 MRT samples (MRT1– 4) using spatial transcriptomics and single-cell sequencing technologies (Methods; Fig. 1A–B). All four were extrarenal MRTs with either SMARCB1 loss (MRT1 and 2) or SMARCA4 loss (MRT3 and MRT4) (Fig. S2A; Table S1).

#### Spatial transcriptomics

Six regions of interest (ROIs) per sample were selected based on T cell infiltration levels, with each ROI containing a CD45 + immune segment and a CD45- tumor segment (Fig. 1B; Methods). In total, 48 segments from 24 ROIs across MRT1–4 were profiled using the Bruker/NanoString GeoMx Digital Spatial Profiler (DSP) Human Whole Transcriptome Atlas (WTA) RNA Assay(Methods). The TIME types (“hot” or “cold”) of selected ROIs were defined based on the proportion of cytotoxic lymphocytes in the immune segments inferred by *in silico* deconvolution tools, CIBERSORTx^23^ and SpatialDecon^23,24^ (Fig. 1C; Table S4–5; Methods). The cytolytic activity (CYT) score^20^ (Methods), which serves as a measure of cytotoxic lymphocyte activity, was positively correlated with cytotoxic lymphocyte infiltration in immune segments (Pearson R = 0.67, p-value = 0.00034; Fig. 1D), supporting the TIME types defined by the deconvolution results.

#### Single-cell multi-omics

Using the 10X Genomics Chromium Single Cell Epi Multiome ATAC + Gene Expression (single-cell Multiome for short) assay, we profiled gene expression and chromatin accessibility in 22,922 nuclei isolated from MRT1–4 and an MRT cell line G401, which we used as a control for SMARCB1 loss when assigning cell types based on SMARCB1 expression (Methods). We combined scRNA and scATAC results from all samples to identify 11 cell clusters (Fig. S1A; Methods). Clusters 1–5 were each composed almost exclusively of cells (> 99.9% of cells) from one sample, while the other six clusters (clusters 6–11) contained cells from multiple samples (Fig. S1B). Notably, clusters 6–11 contained only cells from the four flash-frozen tumor tissues but not from the G401 cell line, compatible with the notion that these clusters were composed of cell types found only in primary tissues. We assigned cell type identity to clusters based on transcriptional and epigenetic features (Fig. S1C-G; Methods). Clusters 1–5 were identified as malignant cell clusters (G401 and MRT1–4), while clusters 6–11 were assigned to fibroblasts, immune cells, endothelial cells, endothelial progenitor cells, adipocytes, and an unidentifiable (“unknown”) cell type (Fig. 1E).

We compared our single cell results to bulk RNA-seq data we generated previously from 65 MRT samples^11^, and to bulk ATAC-seq data we generated in this study for 42 samples from the same cohort (Fig. 1A; Table S1; Methods).

### MRTs express a wide variety of tumor antigens

Malignant rhabdoid tumors share a phylogenetic link with neural crest-derived Schwann cells and are arrested in their differentiation due to SMARCB1 loss^25^. Therefore, tissue-specific and developmental genes may be expressed abnormally in MRTs, triggering an immune response and stimulating T cell infiltration observed in the “hot” ROIs. To test this hypothesis, we compiled a list of 288 tumor antigens (TAs) (Table S3^26^), including cancer-testis antigens and oncofetal genes, and examined their expression levels in MRTs.

**Figure 2:**
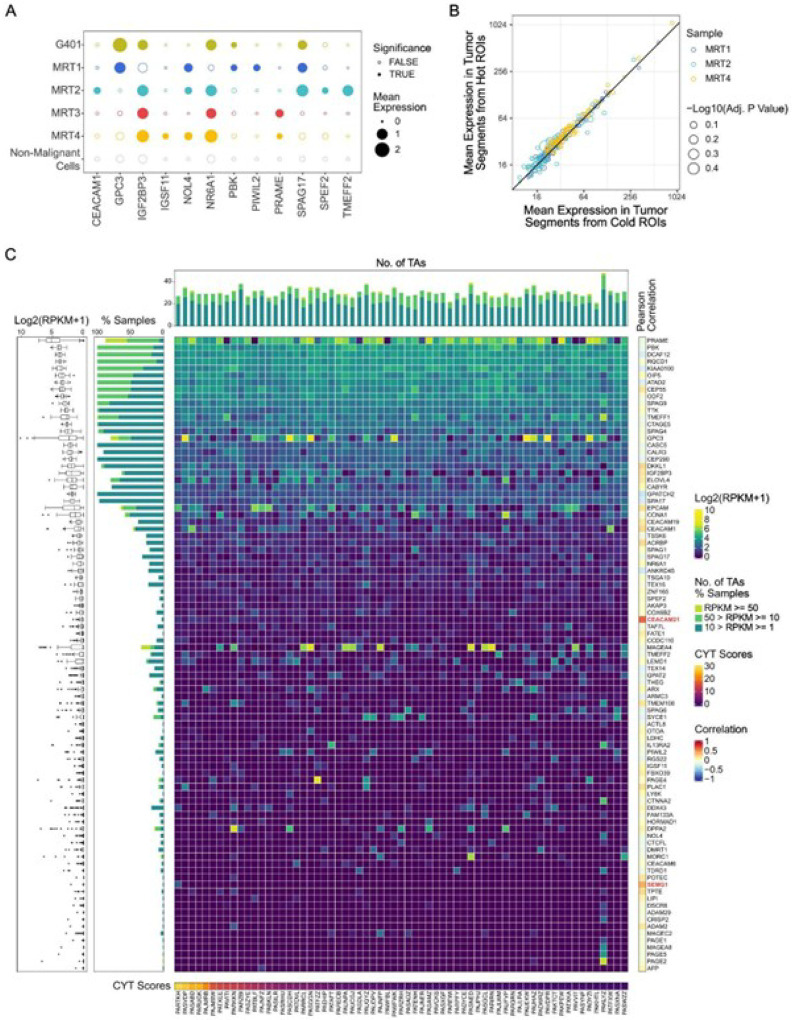
MRTs express a wide variety of tumor antigens (TAs) (See also Figure S2) A) Bubble heatmap depicting mean normalized single cell expression levels of selected TAs in tumor clusters (G401, MRT1–4 clusters) and a non-malignant cell cluster (combined non-malignant cells from MRT1–4). Dot size reflects mean expression levels, with solid dots indicating significantly higher expression in tumor clusters compared to non-malignant cells (one-sided Welch’s t-test, FDR-adjusted p-value < 1e-50). B) Scatter plot showing mean TA expression levels in “hot” versus “cold” tumor segments in each sample profiled using spatial transcriptomics. Each circle represents a detected TA, with color indicating the sample and circle size representing −Log10(FDR-adjusted p-value). All adjusted p-values > 0.05. C) Heatmap depicting expression levels of selected TAs (RPKM >= 1 in at least one MRT sample) across 65 MRTs profiled using bulk RNA-Seq. Columns represent tumor samples, ordered by CYT scores shown beneath the heatmap, while rows represent TAs, sorted by median expression. The boxplot on the left summarizes TA expression across the cohort, while the bar chart between the boxplot and heatmap illustrates the percentage of samples expressing each TA, grouped by expression levels (1–10 RPKM, 10–50 RPKM, and >=50 RPKM). The bar chart on top shows the number of TAs per tumor, categorized by expression levels (1–10 RPKM, 10–50 RPKM, >= 50 RPKM). Pearson correlations between each TA’s expression and CYT scores are shown on the right. TAs with expression levels significantly correlated with CYT scores (FDR-adjusted p < 0.05) are highlighted in red.

First, using single-cell multi-omic data, we compared the mean expression levels of TAs in malignant cell clusters with those in all non-malignant cells from all 4 MRTs (labeled as “non-malignant cells”) (Fig. 2A). We identified 12 TAs with mean expression levels that were 1) above 0.2 normalized counts in at least one tumor cluster and 2) significantly higher than in non-malignant cells (one-side Welch’s t-test, false discovery rate (FDR)-adjusted p-value < 1e-50). We found more than one TA was expressed per tumor, and seven TAs (*GPC3, IGF2BP3, NOL4, NR6A1, PKB, PRAME*, and *SPAG17*) were detected in multiple tumors. The expression of PRAME, a well-known TA frequently overexpressed in various cancers^27–29^, was confirmed using IHC (Fig. S2B).

We then used spatial transcriptomics to compare the mean expression levels of TAs in tumor segments from “hot” and “cold” ROIs within the same sample. Transcripts from 91 TAs were detected above the limit of quantitation (Methods; Table S6) in at least one tumor segment. MRT3 was excluded as all ROIs were categorized as “cold”. Statistical tests indicated that none of these TAs were differentially expressed between “hot” and “cold” tumor segments (two-side Welch t-test, all FDR-adjusted p-values > 0.05; Fig. 2B).

We evaluated the expression levels of TAs in 65 MRT samples profiled using bulk RNA-seq^11^ (Fig. 2C; Methods). 91 TAs (Table S7) were detected with an RPKM (reads per kilobase per million mapped reads) > = 1 in at least one MRT sample. Twenty-three TAs seemed to be broadly expressed (RPKM > = 1 in more than 75% of the MRTs) while others (e.g., *MAGEA4*) showed prominent expression in only a subset of the tumor samples (Fig. 2C). We used the Pearson correlation coefficient to assess the relationship between TA expression and immune cytotoxic activity (measured by CYT score). The CYT score was strongly correlated with cytotoxic T cell markers in TCGA datasets^20^ and was also significantly positively correlated with cytotoxic lymphocyte infiltration (sum of CIBERSORT-inferred CD8 + T cell and activated NK cell fractions) in the MRT bulk RNA-Seq cohort (Pearson R = 0.82, FDR-adjusted p-value = 5.91e-16; Table S8). The expression levels of only two TAs were significantly positively correlated with CYT scores, namely *CEACAM21* (Fig. S2C) and *SEMG1* (Fig. S2D). Other TAs did not show a significant correlation (Pearson R range: −0.29 ~ 0.24). Despite the CYT score correlation, *CEACAM21* was expressed at a low level in our cohort (median RPKM = 0.2054, RPKM > = 1 in 2 samples). Only one sample (PARTKH) had *SEMG1* expression above 1 RPKM, which drove the aforementioned correlation, and when this outlier was removed, the correlation was no longer observed (Fig. S2D). The number of antigens expressed (RPKM > = 1) per tumor sample ranged from 21 to 48 (median 29), which did not correlate with the CYT score (Pearson R = 0.03, p-value = 0.8039).

These findings revealed that MRTs express a wide variety of potential TAs, as shown through single-cell, bulk, and spatial profiling approaches. However, TA expression levels were similar in tumor segments from “hot” and “cold” ROIs, and generally did not correlate with immune cytotoxic activity across MRTs.

### Elevated expression of antigen processing and presentation genes in MRT is associated with a “hot” tumor immune microenvironment

**Figure 3:**
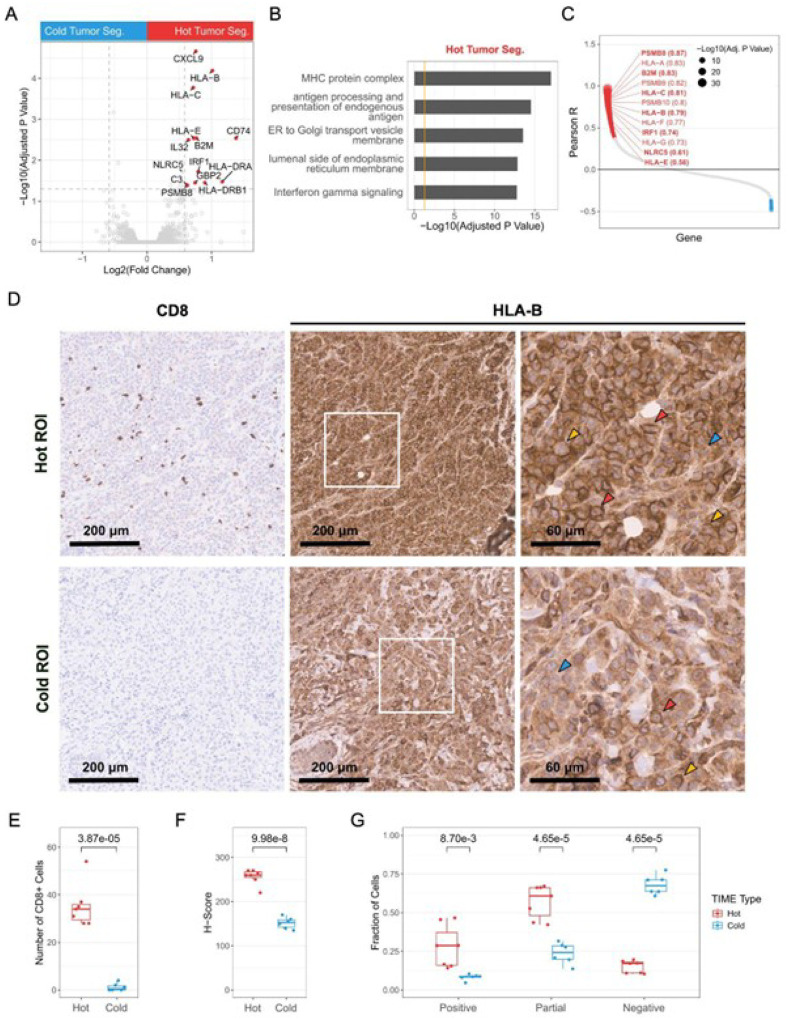
Elevated expression of antigen processing and presentation genes in MRT is associated with a “hot” tumor immune microenvironment A) Volcano plot showing differentially expressed genes between tumor segments from “hot” and “cold” ROIs in the spatial transcriptomic data. Red dots represent genes overexpressed in “hot” tumor segments, with gray dashed lines marking FDR-adjusted p-value = 0.05 and Log2(fold change) = ±Log2(1.5). B) Functional enrichment analysis of genes preferentially expressed (FDR-adjusted p-value < 0.05 and fold-change > 1.5) in tumor segments from “hot” ROIs. The orange line indicates adjusted p-value = 0.05. C) Pearson correlations between gene expression and CYT scores across 65 MRT samples profiled by bulk RNA-Seq. Dot size represents the FDR-adjusted p-value. Statistically significant correlations are shown in red (positive) and blue (negative). Genes involved in antigen processing and presentation are labeled with their Pearson correlation coefficients in parentheses, and APPGs identified in Figure 3A are highlighted in bold. D) Representative IHC images showing the expression of CD8 (left panel) and HLA-B (middle and right panels) in “hot” (top panel) and “cold” (bottom panel) regions from MRT2. The right panels show the enlarged views of the highlighted regions (white squares) from the middle panels. Red, yellow, and blue arrowheads indicate examples of cells categorized as having “Positive” (red), “Partial” (yellow), and “Negative” (blue) membrane HLA-B. E-F) Box plots depicting the number of CD8+ cells (E) and the H-Score for HLA-B staining (F) in the “hot” and “cold” regions of MRT2. P-values were calculated using two-sided Welch’s t-tests and are indicated above the plot. G) Box plots showing manual quantification of “positive”, “partial”, and “negative” membrane HLA-B based on IHC images (examples in Fig. 3D). P-values were calculated using two-sided Welch’s t-tests and are indicated above the plot.

We next identified differentially expressed genes by comparing tumor segments occupying hot ROIs to tumor segments in cold ROIs (Fig. 3A; Table S9; Methods). Genes encoding components of the major histocompatibility complex (MHC) class I components (*HLA-B, HLA-C, HLA-E*, and *B2M*), MHC class I transactivators (*IRF1* and *NLRC5*), and the immunoproteasome subunit *PSMB8* (collectively referred to as “antigen processing and presentation genes”, APPGs) showed significantly higher expression levels in tumor segments within “hot” ROIs, compatible with processing and presentation of antigens. Gene ontology (GO) terms such as “MHC protein complex” and “antigen processing and presentation of endogenous antigen” were associated with the differentially expressed genes (DEGs; FDR-adjusted p-value < 0.05, fold-change > 1.5) in hot ROI tumor segments (Fig. 3B; Table S10). This finding aligns with a previous report that used bulk RNA-seq analysis to show that tumors with high CD8 + T cell populations exhibited elevated expression of antigen processing and presentation genes^15^. The expression of MHC class I genes and immunoproteasome subunits, including the seven APPGs mentioned above, were also significantly (FDR-adjusted p-value < 0.05) positively correlated with CYT scores across the 65 MRTs in the bulk RNA-seq cohort (Fig. 3C).

To confirm these findings, we stained two consecutive FFPE sections of MRT2 with anti-CD8A and anti-HLA-B antibodies (Methods). We selected 13 regions and examined the correlation between the level of infiltrating CD8 + T cells and the level of HLA-B expression in tumor cells across these regions (Fig. 3D and 3E). Tumor cells in regions with higher CD8 + T cell infiltration displayed elevated HLA-B expression, indicated by higher histochemical score (H-scores; Fig. 3F; Methods). These regions also showed a noticeable enrichment of the HLA-B signal at the cell membrane, presenting ring-like staining patterns (Fig. 3D and 3G; Methods). Conversely, tumor cells in regions with low or no CD8 + T cell infiltration exhibited reduced HLA-B levels and lacked enrichment at the cell surface in most cells (Fig. 3D, 3F, and 3G). Overall, these findings revealed an association between higher expression of antigen processing and presentation genes in tumor cells and a “hot” TIME type.

### IRF1 signaling may regulate antigen processing and presentation genes and correlates with TIME types

**Figure 4:**
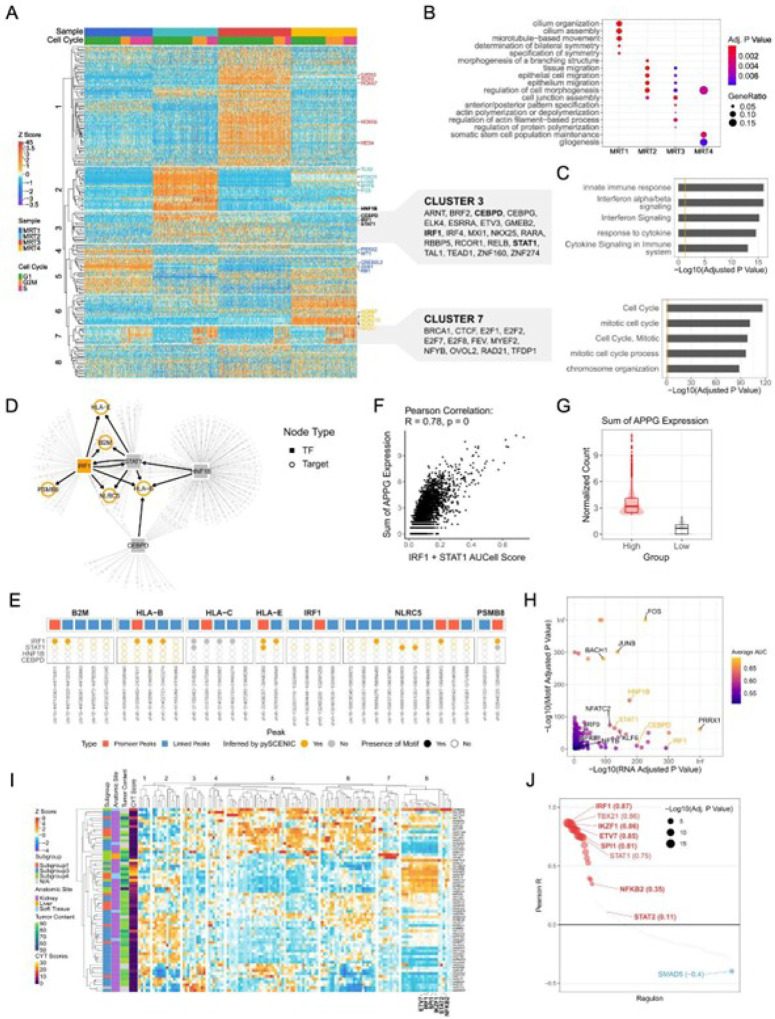
IRF1 signaling is inferred to regulate antigen processing and presentation genes and correlate with TIME types in MRTs (See also Figure S3) A) Heatmap displaying regulon activity scores (z-scores derived from AUCell scores) in malignant cells profiled using single-cell multi-omics. Columns represent individual cells, and rows represent regulons. Top annotations indicate cell sources and inferred cell cycle phases (based on the expression of G2/M and S phase markers from the scRNA-Seq data). The top 5 regulons with the highest regulon-specific score (RSS) in each tumor cluster (MRT1–4, Fig. S3A) are labeled on the right and colored by sample. Regulons inferred to regulate APPG expression are highlighted in bold. B) Functional enrichment analysis of TFs and targets in regulons with RSS greater than 0.5 in each MRT. C) Functional enrichment analysis of TFs and their targets within regulons in clusters 3 and 7 (orange line marks adjusted p-value = 0.05). D) Transcriptional regulatory network based on regulons inferred from single-cell multi-omic data using pySCENIC. TFs and targets are shown as solid squares and open circles, respectively. The APPGs are highlighted in orange. E) Visualization of TF motifs within the promoters and inferred linked peaks of APPG. The top panel shows the promoter (red) and linked peaks (blue). In the bottom panel, solid and open circles represent the presence or absence of motifs in each peak, respectively. Circles are colored orange if the APPGs are inferred to be regulated by the TFs by pySCENIC. F) Scatter plot showing the correlation between the combined *IRF1* and *STAT1* regulon activities (sum of their AUCell scores) and the total APPG expression levels. G) Box and violin plots showing the distribution of total APPG expression in “APPG high” and “APPG low” groups. H) Scatter plot showing TFs with higher expression and enriched motif accessibility in the “APPG high” group compared to the “APPG low” group. The x- and y-axes represent the adjusted p-values for gene expression and motif accessibility enrichment, respectively. Only TFs with adjusted p-values < 0.05 for both are shown. Dots are colored by the mean of AUC (area under the curve) values calculated from gene expression and motif accessibility data, reflecting the strength as markers. I) Heatmap displaying regulon activity scores (z-scores derived from AUCell scores) derived from the bulk RNA-Seq data. Each row represents an MRT sample, and each column represents a regulon. Left annotations indicate methylation-based subgroups^15^, anatomic sites, tumor contents, and CYT scores. Regulons inferred to regulate the expression of *HLA-A/B/C/E/F/G, B2M, IRF1, NLRC5*, and *PSMB8/9/10* are labeled. J) Pearson correlations between regulon regulon activity (AUCell) scores and CYT scores across 65 MRT samples profiled by bulk RNA-Seq. Dot size represents the FDR-adjusted p-value. Statistically significant correlations are shown in red (positive) and blue (negative). Regulons inferred to regulate the expression of *HLA-A/B/C/E/F/G, B2M, IRF1, NLRC5*, and *PSMB8/9/10* are highlighted in bold, with the Pearson correlation coefficients labeled in parentheses.

The observation that multiple genes involved in antigen processing and presentation were more highly expressed in the “hot” tumor segments motivated us to investigate the signaling networks regulating this pathway in tumor cells. We used pySCENIC^30,31^ and single cell expression data to infer “regulons”, defined as gene sets regulated by the same transcription factor (Methods). In total, 217 regulons were predicted to be active in at least a subset of tumor cells (Table S11). MRT1–4 each displayed different regulon activation patterns (Fig. 4A and S3A; Table S11) linked to different biological processes (Fig. 4B; Table S12). As expected, pySCENIC identified the activation of regulons associated with cell cycle regulation (cluster 7) in cells inferred to be in the G2M and S phases (Fig. 4A and 4C; Table S13; Methods). Notably, regulons in cluster 3 were associated with immune response pathways, such as “interferon signaling” and “antigen processing and presentation of endogenous peptide antigen via MHC class I” (Fig. 4A and 4C; Table S13).

Four regulons (*IRF1, STAT1, HNF1B*, and *CEBPD* regulons) were inferred to control the expression of at least one APPG (Fig. 4D; Table S14). Analysis of gene expression and chromatin accessibility data coprofiled from the same nuclei revealed open chromatin regions correlated with the expression of each APPG (referred to as “linked peaks”, Methods). *IRF1* and *STAT1* motifs were present in the promoter and/or linked peaks of several APPGs, indicating they may directly regulate these APPGs (Fig. 4E). In contrast, *HNF1B* and *CEBPD* motifs were not found in any peaks (Fig. 4E). The inferred activity of *IRF1* and *STAT1* regulons were positively correlated with the sum of normalized APPG expression in tumor cells (Pearson R = 0.78, p-value = 0; Fig. 4F). When cells were divided into “APPG high” and “APPG low” groups based on total APPG expression (Fig. 4G; Methods), both *IRF1* and *STAT1* showed greater inferred activity (higher transcription factor (TF) expression and greater motif enrichment) in the “APPG high” group (Fig. 4H, Table S15; Methods). Other TFs, for example, *IRF9, NFKB1*, and *NFYB*, which are known to regulate the expression of MHC class I genes and immunoproteasome subunits^32^, also exhibited greater inferred activity in the “APPG high” group, further supporting enhanced antigen processing and presentation in these cells.

To validate these findings in a larger cohort and examine the correlation between regulon activity and TIME types (indicated by CYT scores) across MRT samples, we performed regulon analysis using the 65 bulk RNA-seq datasets. We identified six TFs (*IRF1, STAT2, SPI1, IKZF1, ETV7*, and *NFKB2*) potentially regulating genes involved in antigen processing and presentation (*HLA-A/B/C/E/F/G, B2M, IRF1, NLRC5*, and *PSMB8/9/10*) (Fig. 4I, S3B; Table S16). Hierarchical clustering grouped these regulons into cluster 8, co-activated in a subset of MRTs (Fig. 4I). Activities of all but the *STAT2* regulons were significantly positively correlated with the CYT scores (Fig. 4J). *IRF1* was again inferred to regulate APPG expression, confirming our single-cell results, and showed the highest correlation with CYT scores. Functional enrichment analysis revealed that regulons in cluster 8 were involved in interferon-gamma signaling and antigen processing and presentation (Fig. S3C; Table S17). By integrating bulk ATAC-seq data, available for 42 samples, we identified promoter and linked peaks for each target gene and examined TF motifs within these peaks for genes inferred to be regulated by the corresponding TF (Methods). Motifs of all TFs were confirmed in the promoter and/or linked peaks of several predicted targets (Fig. S3D).

Together, our analysis indicated higher *IRF1* expression in tumor segments in “hot” ROIs and a correlation between *IRF1* signaling and CYT scores across MRTs. Such variation in *IRF1* signaling could potentially influence APPG expression levels in tumor cells, affect their antigen processing and presentation capabilities and ultimately impact tumor immunity and TIME type.

### The skeletal muscle development program is upregulated in a subset of MRTs with high CD8 + T cell infiltration

**Figure 5:**
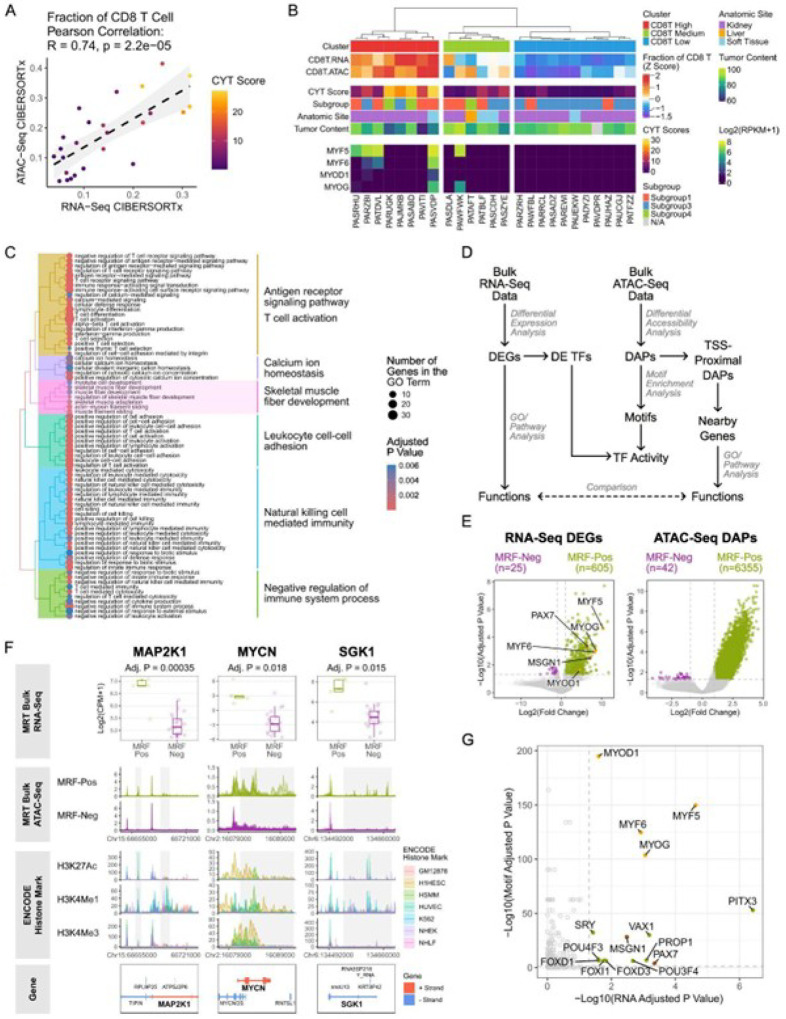
The skeletal muscle development program is upregulated in a subset of MRTs with high CD8 T cell infiltration (See also Figure S3) A) Scatter plot showing the CIBERSORTx-inferred CD8+ T cell fractions based on bulk RNA-Seq and bulk ATAC-Seq data. Dots are colored by CYT scores. The grey band shows the 95% confidence interval. B) The top panel shows hierarchical clustering based on CIBERSORTx-inferred CD8+ T cell fractions from bulk RNA-seq and ATAC-seq data (shown as z scores). The middle panel includes annotations for CYT scores, methylation-based subgroups^15^, anatomic sites, and tumor content. The bottom panel displays the expression levels of the four myogenic regulatory factors (MRFs). C) Hierarchical clustering of the top 75 enriched GO terms (ranked by adjusted p-values) associated with genes preferentially expressed (FDR-adjusted p-value < 0.05, fold-change > 2; Table S16) in the “CD8T High” group (Fig. 5B). Dot size indicates the number of genes preferentially expressed in the ‘“CD8T High” group within each GO term, and dot color represents the adjusted p-values. D) Integrative analysis pipeline for matched bulk RNA-seq and ATAC-seq data. DEGs: differentially expressed genes; DAPs: differentially accessible peaks, DE TFs: differentially expressed transcription factors. TSS: transcription start site. E) Volcano plots showing DEGs (left plot) and DAPs (right plot) between MRF-positive (green dots) and MRF-negative MRTs (purple dots). Gray dashed lines mark FDR-adjusted p-value = 0.05 and Log2(fold change) = ±Log2(2). The four MRFs are highlighted in orange. PAX7 and MSGN1 are highlighted in brown. F) Examples of DEGs with TSS-proximal DAPs. The top panel shows box plots of normalized gene expression between MRF-positive and negative groups. Below, normalized ATAC-seq signals and ENCODE histone marks^36,82–84^ (H3K27Ac, H3K4Me1, and H3K4Me3) from seven cell lines (GM12878, a lymphoblastoid cell line; H1HESC, H1 human embryonic stem cell line; HSMM, human skeletal muscle myoblasts; HUVEC, human umbilical vein endothelial cells; K562, a human erythroleukemic cell line; NHEK, normal human epidermal keratinocytes; and NHLF, normal human lung fibroblasts) are displayed. Gray boxes highlight regions with increased chromatin accessibility in MRF-positive samples. Gene models are shown in the bottom panel. G) Scatter plot showing TFs with higher expression and enriched motif accessibility in the MRF-positive MRTs compared to MRF-negative MRTs. The x- and y-axes represent the FDR-adjusted p-values for gene expression and motif accessibility enrichment, respectively. Gray dashed lines mark adjusted p-value = 0.05. The four MRFs are colored in orange. PAX7 and MSGN1 are colored in brown.

To further investigate transcriptional programs upregulated in tumors with “hot” TIME, we used CIBERSORTx to infer immune cell compositions for the 42 MRTs with matched bulk RNA-seq and ATAC-seq data (Table S18–19; Methods). We focused on samples where ATAC and RNA-seq both estimated a similar proportion of CD8 + T cells (Fig. 1A; Methods). Twenty-five samples met these criteria and were grouped into “CD8T High,” “CD8T Medium,” and “CD8T Low” clusters using hierarchical clustering based on the inferred CD8 + T cell fractions (Fig. 5B; Methods). Differential expression analysis identified 189 DEGs (FDR-adjusted p-value < 0.05, fold-change > 2) enriched in the “CD8T High” compared to “CD8T Low” groups (Table S20). In addition to immune response-related genes, the “CD8T High” group preferentially expressed skeletal muscle development genes (Fig. 5C; Table S21), including myogenic regulatory factors (MRFs) such as *MYF6, MYOD1*, and *MYOG*^33^ (Table S20). This was attributed to a greater proportion of MRF-positive samples (samples have at least one MRF with > = 1 RPKM) in the “CD8T High” group (one-sided Fisher’s exact test, p-value = 0.02345; Fig. 5B). Compared to the MRF-negative MRTs (n = 20), the MRF-positive MRTs (n = 5) exhibited 605 DEGs and 6,355 differentially accessible peaks (DAPs), including 333 peaks within 1,000 bp of transcription start sites (TSS-proximal DAPs) (Fig. 5D–E; Table S22–23). Both the DEGs and genes with TSS-proximal DAPs in the MRF-positive samples were associated with skeletal muscle development (Table S24). Seventy-eight DEGs in MRF-positive samples had at least one TSS-proximal DAP (Table S25). Examples include *MAP2K1*, a key component of mitogen-activated protein kinase (MAPK) pathway, which showed higher expression with additional open chromatin regions at potential regulatory sites in MRF-positive MRTs (Fig. 5F). Inhibition of *MAP2K1/2* has been shown to reduce proliferation and promote apoptosis in AT/RT cell lines^34^. While *MYC* expression remains similar between MRF-positive and negative groups (FDR-adjusted p-value = 0.52), *MYCN*, which plays a critical role in promoting cell proliferation during embryonic development^35^, appears to be more highly expressed in MRF-positive MRTs with expanded chromatin accessibility (Fig. 5F). Interestingly, the human embryonic stem cell line (H1HESC) and human skeletal muscle myoblasts (HSMM) exhibit stronger H3K27Ac^36^ and H3K4Me3^36^ signals across the *MYCN* region compared to more differentiated cell lines (Fig. 5F). *SGK1*, a serine/threonine kinase in the PI3K signaling pathway, is also upregulated in MRF-positive MRTs, accompanied by increased chromatin accessibility at potential enhancer regions (Fig. 5F). All four MRFs (*MYF5, MYF6, MYOG*, and *MYOD1*) showed higher expression levels and enriched motifs in the MRF-positive samples, suggesting generally increased MRF activity (Fig. 5D, 5G; Table S26). Additionally, *PAX7*, which participates in embryonic skeletal muscle development and is required for the specification of satellite cells (muscle stem cells)^37,38^, and *MSGN1*, a key regulator in the development of the paraxial mesoderm that gives rise to skeletal muscles^39^, were inferred to be active in the MRF positive samples (Fig. 5D, 5G; Table S26). Using single-cell multi-omic data from MRT1–4, we mapped MRF expression to malignant cells (Methods). *MYF6* exhibited elevated expression levels and greater motif enrichment in MRT2 tumor cells (Fig. S3E), consistent with the higher *MYF6* regulon specificity scores (RSS, representing how specific a regulon is to a particular cell population^31^) observed in these cells (Fig. S3A). These findings revealed that a subset of MRTs with “hot” TIME display a distinct transcriptional profile characterized by enhanced skeletal muscle development programs.

### The M1/M2 macrophage ratio is correlated with cytotoxic lymphocyte infiltration

**Figure 6:**
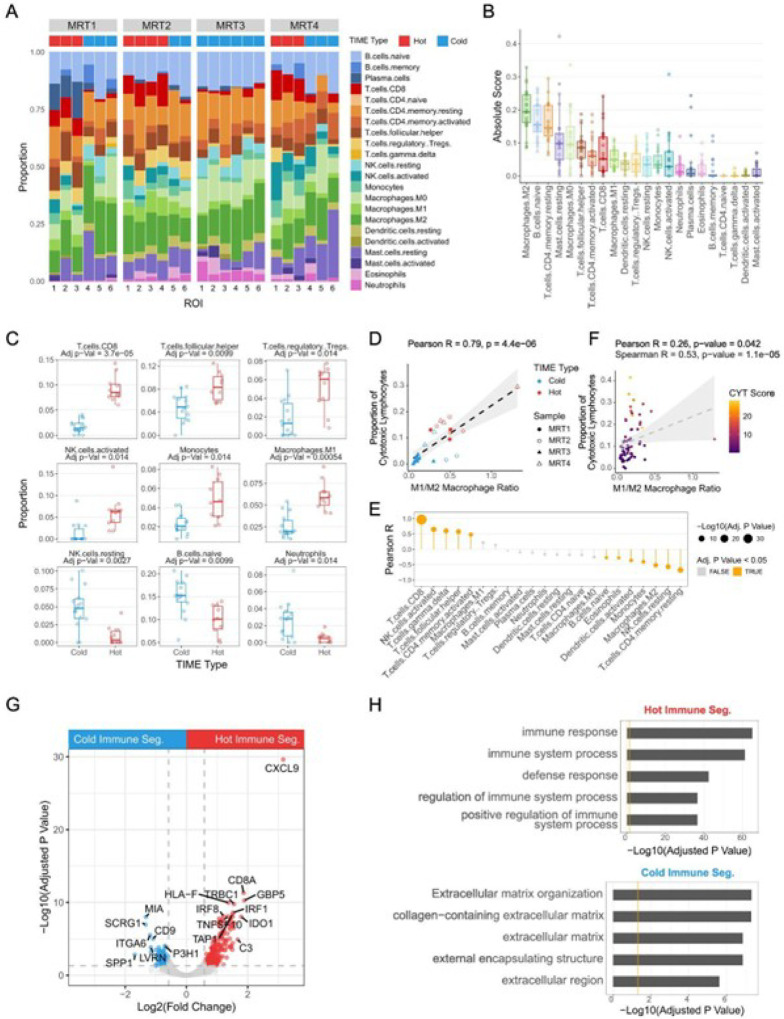
The M1/M2 macrophage ratio is positively correlated with cytotoxic lymphocyte infiltration (See also Figure S4) A) Stacked bar plot showing the CIBERSORTx-inferred relative abundance of immune cell populations in the immune segments from the 24 ROIs profiled by spatial transcriptomics, with the TIME type of each ROI labeled above. B) Box plot showing the absolute scores of immune cell types inferred by CIBERSORTx from immune segments in the spatial transcriptomic data. Each dot represents one of the 24 immune segments from the 24 ROIs across the four MRT samples. C) Box plots showing differences in immune cell types inferred by CIBERSORTx between immune segments from “hot” and “cold” ROIs. P-values were calculated using two-sided Welch t-tests, with FDR correction for multiple comparisons. D) Scatterplot showing the correlation between M1/M2 macrophage ratio and the proportion of cytotoxic lymphocytes in the tumor microenvironment. The grey band shows the 95% confidence interval. E) Lollipop plot showing correlations between the fraction of cytotoxic lymphocytes (the sum of CD8 T cell and activated NK cell fractions) and the fraction of other immune cell types inferred by CIBERSORTx using bulk RNA-Seq data. The FDR was used to correct for multiple comparisons. F) Scatterplot showing the correlation between M1/M2 macrophage ratio and proportion of cytotoxic lymphocytes inferred by CIBERSORTx using bulk RNA-Seq data. The grey band shows the 95% confidence interval. G) Volcano plot showing differentially expressed genes between immune segments from “hot” and “cold” ROIs in the spatial transcriptomic data. Red and blue dots represent differentially expressed genes in “hot” and “cold” immune segments, respectively, with gray dashed lines marking FDR-adjusted p-value = 0.05 and Log2(fold change) = ±Log2(1.5). H) Functional enrichment analysis of genes preferentially expressed (FDR-adjusted p-value < 0.05 and fold-change > 1.5) in “hot” (top) and “cold” (bottom) immune segments. The orange line indicates adjusted p-value = 0.05.

Next, we characterized non-malignant cells in MRT, using spatial transcriptomic data and CIBERSORTx to infer and compare immune cell populations in “hot” and “cold” immune segments (Methods; Fig. 6A; Table S4). As reported from analysis of bulk RNA-seq data^14^, M2-like macrophages were the most abundant population, followed by resting memory CD4 + T cells and naive B cells (Fig. 6B). We then identified cell types enriched in “hot” and “cold” immune segments (two-sided Welch t-tests, Methods; Fig. 6C and S4A). In addition to CD8 + T cells and activated NK cells, monocytes, M1 macrophages, T follicular helper cells (Tfh), and regulatory T cells (Treg) were significantly enriched in the “hot” immune segments. In contrast, higher proportions of resting NK cells, naive B cells, and neutrophils were found in immune segments from “cold” ROIs. M2-like macrophages showed a non-significant trend toward higher abundance in “cold” regions (Fig. S4A), but the M1/M2 macrophage ratio correlated significantly with cytotoxic lymphocyte infiltration (Pearson R = 0.79, p-value = 4.433e-06; Fig. 6D).

To validate our observations in a larger cohort, we performed *in silico* cell type deconvolution using CIBERSORTx and 65 bulk RNA-seq datasets^11^ (Table S18; Methods) and calculated the Pearson correlation coefficients between the fraction of each cell type and the fraction of cytotoxic lymphocytes (sum of CD8 + T cell and activated NK cell fractions; Fig. 6E, Table S27). In addition to CD8 + T cells and activated NK cells, gamma delta T cells (γδ T cells), T follicular helper cells (Tfh), and activated memory CD4 + T cells were significantly positively correlated with cytotoxic lymphocyte fractions across patients. M1 macrophages and regulatory T cells (Treg) also showed positive, though not significant, correlations. The cell types significantly negatively correlated with the cytotoxic lymphocyte fraction included resting memory CD4 + T cells, resting NK cells, M2 macrophages, monocytes, activated dendritic cells, eosinophils, and naive B cells. Furthermore, the M1/M2 macrophage ratio was significantly positively correlated with cytotoxic lymphocyte levels (Pearson R = 0.26, p-value = 0.042 with outlier, Fig. 6F; Pearson R = 0.45, p-value = 0.00023 without outlier, Fig. S4B). Overall, these findings reveal distinct immune cellular compositions associated with different TIME types and support a relationship between the M1/M2 macrophage ratio and cytotoxic lymphocyte levels observed in the spatial transcriptomic data.

Next, we performed differential gene expression analyses using spatial transcriptomic data to identify genes and pathways associated with different TIME types. This revealed 245 and 126 DEGs (FDR-adjusted p < 0.05, fold-change > 1.5) in immune segments from “hot” and “cold” ROIs, respectively (Fig. 6G; Table S9; Methods). “Hot” immune segments exhibited higher expression of genes encoding T cell receptors (*CD8A, CD3E, TRAC*, and *TRBC1*), chemokines (*CXCL9, CXCL10, CXCL13, CCL5*, and *CCL19*), interferon signaling factors (*IRF1, IRF8, STAT1*, and *GBP1*), immunoproteasomes (*PSMB8/9/10*), MHC complexes (*HLA-A/B/C/E/F/DQA1/DQA2/DQB1/DPA1/DPB1/DRA/DRB1*), and MHC transactivators (*NLRC5* and *CIITA*). *LYZ*, the gene encoding lysozyme, showed a positive correlation with M1-like macrophage and cytotoxic T cell numbers^40^. It was more highly expressed in the “hot” immune segments, aligning with the higher M1/M2 ratio and T cell levels in these regions (Table S9). Pathway analysis revealed that genes overrepresented in “hot” immune segments were involved in immune response and lymphocyte activation (Fig. 6H; Table S10; Methods). In contrast, genes overexpressed in “cold” immune segments were associated with extracellular matrix organization (Fig. 6H; Table S10). Several genes in cold segments (e.g. *SPP1, CD9*, and *SCRG1*) were previously implicated in M2-like macrophage polarization and anti-inflammatory activities^41–44^ (Fig. 6G; Table S9).

### Multi-omic characterization of MRT-associated macrophages revealed genes and pathways underlying M2-like phenotype

**Figure 7:**
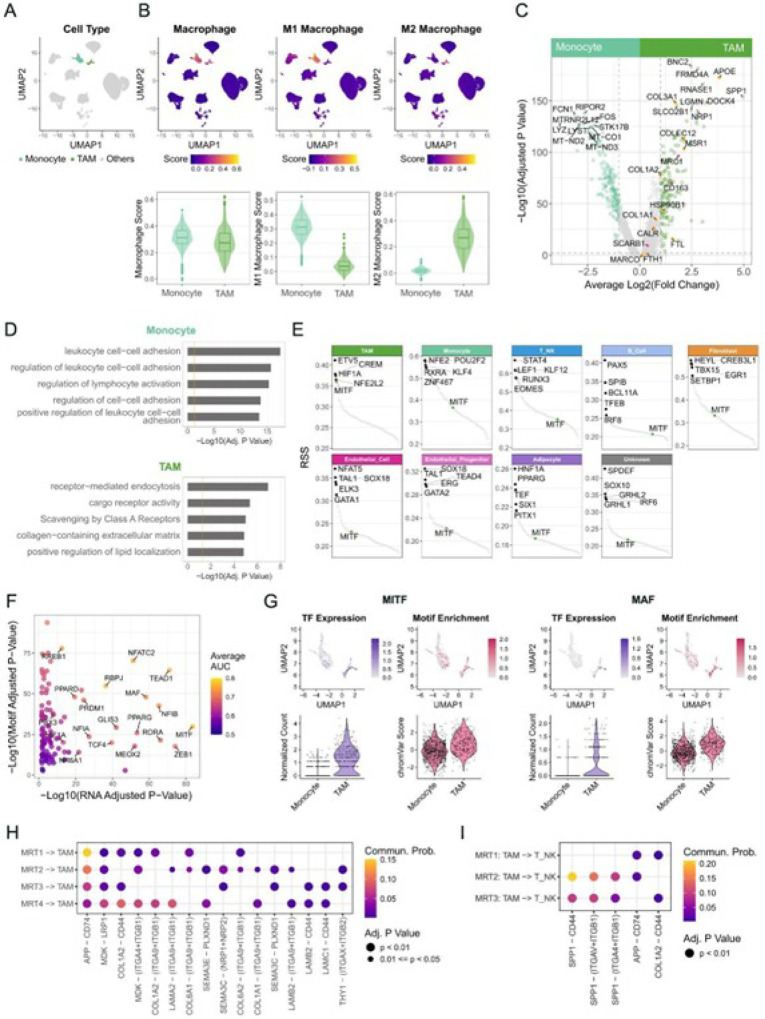
Multi-omic characterization of MRT-associated macrophages revealed genes and pathways underlying M2-like phenotype (See also Figures S5 and S6) A) UMAP visualizations of PBMC and MRT clusters profiled by single-cell multi-omic assay (Fig. S5B). The monocyte and TAM clusters are colored. B) The overall expression scores of macrophage, M1 macrophage, and M2 macrophage signatures are colored on UMAP (top) and presented as box and violin plots (bottom). C) Volcano plot showing differentially expressed genes between monocyte and TAM clusters profiled by single-cell multi-omic assay. The light teal and green dots represent differentially expressed genes in monocytes and TAM clusters, respectively, with gray dashed lines marking FDR-adjusted p-value = 0.05 and Log2(fold change) = ±Log2(1.5). Orange dots indicate genes in the gene set “Scavenging by Class A Receptors” (Reactome: R-HSA-3000480), while pink dots indicate additional scavenger receptor genes. D) Functional enrichment analysis of genes preferentially expressed (FDR-adjusted p-value < 0.05 and fold-change > 1.5) in monocyte (top) and TAM (bottom) clusters. The orange line indicates adjusted p-value = 0.05. E) Regulon-specific score (RSS) plot depicting the specificity of regulons in each cell type. *MITF* regulon is highlighted in green in all subplots. F) Scatter plot illustrating TFs with higher expression and enriched motif accessibility in the TAM cluster compared to the monocyte cluster. The x- and y-axes represent the adjusted p-values for gene expression and motif accessibility enrichment, respectively. Only TFs with adjusted p-values < 0.05 for both are shown. Dots are colored by the mean of AUC values calculated from gene expression and motif accessibility data, reflecting the strength as markers G) Comparing the gene expression (colored in purple) and motif accessibility (colored in pink) of *MITF* and *MAF* in the monocyte and TAM clusters, visualized on UMAP (top) and as violin plots (bottom). H-I) Dot heatmaps showing inferred cell-cell communications between tumor cells and TAMs (H) and between TAMs and T cells (I) using single-cell multi-omic data. MRT4 was excluded from the TAM-T cell interaction analysis due to insufficient T cells. Each row of dots represents the inferred interactions between the indicated cell types. Each column represents a ligand-receptor pair. Only ligand-receptor pairs identified in multiple samples were displayed. The dot color shows communication probabilities reported by CellChat.

To identify genes and signaling pathways up-regulated in the TAMs, we compared TAM single-cell profiles to single-cell profiles from peripheral blood mononuclear cells (PBMCs) of a healthy donor (Methods). We merged the MRT and the PBMC single-cell gene expression and ATAC datasets and identified 14 clusters (Fig. S5; Methods). These included a “TAM” cluster with 342 cells and a “monocyte” cluster containing 32 cells from MRT1–4, and 815 cells from the PBMC dataset (Fig. 7A). Using macrophage signature genes (Table S2), we found that monocyte cluster cells exhibited an M1-like phenotype, while the TAM cluster cells showed higher M2 macrophage signature scores (Fig. 7B).

Differential gene expression comparisons of these two clusters revealed the monocyte cluster expressed mitochondrial respiratory chain genes (e.g. *MT-CO1/2/3, MT-ND1/2/3/4/5, MT-CYB*, and *MT-ATP6/8*) and genes involved in regulating multiple aspects of the immune system, such as leukocyte cell-cell adhesion, regulation of T cell activation, and lymphocyte proliferation (Fig. 7C–D; Table S28–29). *LYZ* also showed higher expression in the monocyte cluster (expressed in 92.8% of cells in the monocyte cluster and 15.5% of cells in the TAM cluster; Table S28). In contrast, the TAM cluster differentially expressed genes involved in endocytosis and scavenger receptor pathways (Fig. 7C–D; Table S28–29). Notably, *SPP1*, an immune checkpoint gene suppressing T cell activation^41^, was strongly expressed in the TAM cluster (71.1% of cells) compared to the monocyte cluster (0.7% of cells) (Fig. 7C; Table S28). As noted earlier, *SPP1* was more highly expressed in “cold” immune segments in the spatial transcriptomics data, consistent with the notion that these regions tend to have a higher proportion of M2-like macrophages.

We next sought to identify transcriptional regulators potentially underpinning these TAM gene expression patterns. Regulon analysis identified cell-type-specific regulatory networks for each non-malignant cell type, including those previously reported, for example, *PPARG* in adipocytes^45^; *PAX5* regulon in B cells^46,47^; *EOMES*, and *RUNX3* regulons in T cells and NK cells^48–53^; and *SOX18* in endothelial cells^54^ (Fig. 7E; Table S30). *CREM* and *NFE2L2* have been linked with the anti-inflammatory phenotype of M2-like macrophages^55,56^, and their regulons indeed displayed high RSS in M2-like TAMs. A *HIF1A* regulon also showed high RSS, consistent with lower expression levels of mitochondrial respiratory chain genes in the TAM cluster compared to the monocyte cluster (Fig. 7C; Table S28). Interestingly, the *MITF* regulon showed high specificity in TAMs but was low in other non-malignant cell types. *MITF*, a master regulator of melanogenesis and a melanoma oncogene, has also been reported to regulate the immunosuppressive functions of myeloid-derived suppressor cells (MDSCs) in a mouse model^57^. We identified 106 TFs with elevated inferred activity (higher gene expression and greater motif enrichment) in the TAM cluster compared to the monocyte cluster (Fig. 7F, Table S31). These included TFs known to promote an M2-like phenotype and are critical for TAM-mediated immunosuppressive functions, such as *MAF*^58^ (Fig. 7G). Notably, *MITF* also exhibited greater inferred activity in the TAM cluster (Fig. 7G), consistent with the *MITF* regulon’s higher specificity in TAMs (Fig. 7E).

Taken together, the single-cell multi-omic data confirmed a stronger M2 signature in MRT TAMs and revealed regulatory networks (e.g. the *MITF* regulon) that potentially play roles in their functions. Our analyses also revealed the upregulation of *SPP1* in M2-like TAMs, which may suppress T cell activation^41^.

### Inference of cell-cell interaction identified potential crosstalk that may shape the tumor immune microenvironment

To explore potential interactions in tumors, we used CellChat^59^ (Methods) and single-cell multi-omics data to infer cell-cell interactions in MRT1–4 (Fig. S6A-B). We identified 360 significant (p-value < 0.05) ligand-receptor pairs from 72 pathways, including 198 pairs between tumor and non-malignant cells that span 44 pathways (Fig. S6C; Table S32–33). Focusing on the potential influence of tumor cells on macrophages, we identified 37 ligand-receptor interactions, with *APP-CD74* and *MDK-LRP1* consistently observed across all four samples (Fig. 7H). Interestingly, a study has shown that *MDK* is upregulated in gall bladder cancer with *ErbB* pathway mutations and promotes the differentiation of immunosuppressive macrophages by interacting with *LRP1* on TAM^60^. Several interactions between TAMs and T cells were also predicted, including the *SPP1-CD44* interaction in MRT2 and MRT3 (Fig. 7I). Overall, these findings may reveal potential tumor-macrophage and macrophage-T cell interactions that could lead to an immunosuppressive TIME and complicate the development of effective treatment approaches.

## Discussion

MRT biology must be better understood to create more effective therapeutic strategies. Chromatin modifiers, kinases, cell cycle regulators, and signaling pathways have emerged as potential therapeutic targets in both preclinical studies and clinical trials^5,61^. Despite the low tumor mutational burden, the observed heterogeneity in immune cell infiltration in rhabdoid tumors has sparked interest in investigating immunotherapy as a potential treatment avenue^5,61^. An ongoing Phase II trial (NCT04416568) is evaluating the safety and effectiveness of combined PD-1 and CTLA-4 blockade in *SMARCB1*-negative tumors, including MRT. Motivated by these developments, we sought to explore the heterogeneity in TIME types, which is relevant for developing effective therapeutic strategies.

Our study revealed a wide range of TA expression profiles in MRT, lending support to the investigation of TA-targeted immunotherapies as a treatment option. For example, the cancer-testis antigen encoded by *PRAME* was expressed in a substantial proportion of MRT. Overexpression of *PRAME* has been reported in many pediatric and adult cancers^27–29^, which has ignited research focused on immune-based interventions that specifically target *PRAME* in several cancer contexts^62^. Another gene, *MAGEA4*, exhibited elevated expression in some MRTs. Currently, a targeted treatment using genetically modified autologous T cells for multiple *MAGEA4*-positive solid cancers is undergoing phase I clinical trials (NCT03132922) and has shown promising results^63^.

**Figure 8:**
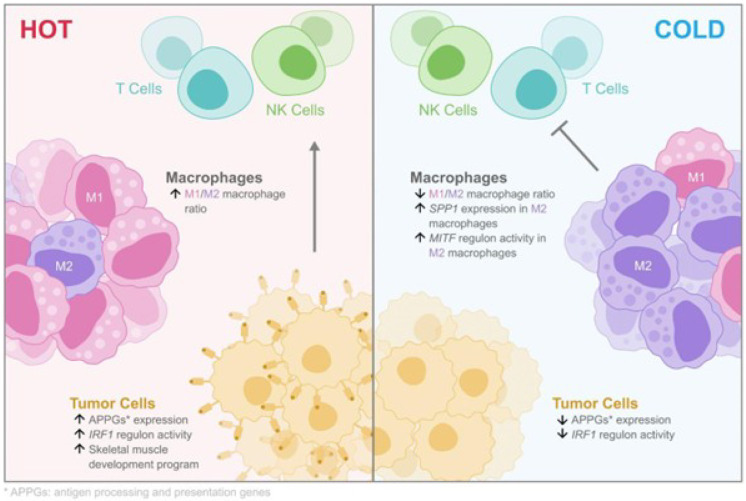
A simplified model of genes and pathways associated with different TIME types in MRT

Interestingly, the expression of most TAs did not correlate with the TIME types, consistent with previous observations^14^, indicating the possible presence of additional factors influencing immune response. Our analyses uncovered intra- and inter-tumor heterogeneity in the expression of genes involved in antigen processing and presentation pathways (Fig. 8). We also observed variability in the inferred activity of the *IRF1* regulatory network, which was predicted to regulate these APPGs (Fig. 8). Both APPGs and inferred *IRF1* regulon activity strongly correlated with cytotoxic lymphocyte infiltration and/or activity. Therefore, APPG expression levels and *IRF1* signaling activity may help stratify MRT patients for immunotherapy.

Notably, IFNγ treatment can enhance the expression of HLA genes in MRT cell lines^64^. Several clinical trials are underway to evaluate the efficacy of IFNγ as a standalone treatment or in combination with other anticancer drugs^65^ highlighting the potential of cytokines as an intervention for promoting immune recognition and response.

The upregulation of skeletal muscle developmental programs were observed in five MRTs and appeared correlated with higher CD8 + T cell infiltration levels (Fig. 8). This is consistent with a recent study that revealed that overexpression of muscle development-related genes are associated with better survival outcomes in a subgroup of MRTs^66^. Additionally, we identified genes that may be involved in tumor growth in MRF-positive MRTs and may present novel therapeutic potential. For example, the MAPK pathway is frequently activated in AT/RTs^67^. Binimetinib (MEK162), a *MAP2K1/2* inhibitor, has been shown to reduce the growth of AT/RT cell lines *in vitro* and in flank xenografts^34^. This drug is FDA-approved for treating *BRAF*-mutant melanoma^68^ and non-small cell lung cancer^69^ in combination with the *BRAF* inhibitor encorafenib and is undergoing Phase II clinical trials for pediatric low-grade gliomas and other *Ras/Raf/ERK* pathway-activated tumors (NCT02285439).

Our analyses revealed immune cell populations associated with distinct TIME phenotypes both within and across tumors, and identified a significant positive correlation between the M1/M2 macrophage ratio and the infiltration of cytotoxic lymphocytes in MRTs (Fig. 8). Studies have reported that the subtypes of TAMs, particularly the M1/M2 macrophage ratio, is a better prognostic predictor compared to overall TAM densities^70–74^. Furthermore, emerging evidence reveals the key roles of macrophage differentiation in treatment resistance and improved responses with macrophage manipulations. M2-like TAMs also contribute to immune checkpoint blockade (ICB) therapy failure and are associated with suboptimal outcomes of CAR-T cell therapy in mouse models^75–77^. These findings underscore the potential of targeting TAMs as a promising adjunct to conventional immunotherapies. Encouragingly, agents targeting macrophages are undergoing clinical trials for cancer treatment^78^.

By employing a suite of genomics approaches, our study discovered pathways and regulatory networks that exhibit greater specificity and enhanced activity in M2-like macrophages compared to M1-like monocytes (Fig. 8). For example, our results highlighted *MITF* as an important TF gene in TAMs from MRT patients. A recent study used mouse models to demonstrate that *MITF* is upregulated in tumor-associated monocytic MDSCs and contributes to their immunosuppressive function^57^. Remarkably, suppression of MITF expression led to reduced tumor size and enhanced T-cell activity^57^. Compounds such as ML329 and CH5552074 have been reported to inhibit the activity of the *MITF* pathway^79,80^, offering exciting possibilities for the development of innovative therapeutic strategies that target *MITF*-expressing TAMs. We also found that *SPP1* is upregulated in M2-like TAMs, which may suppress T cell activation^41^ through the inferred *SPP1-CD44* interaction between TAMs and T cells. This suggests that *SPP1* could be a potential target for modulating the TIME in MRT.

### Limitations of the study

Our study has limitations that should be acknowledged. First, 4 samples were analyzed using single-cell multi-omic and spatial transcriptomic technologies, all from soft tissue, due to the scarcity of primary MRT samples (in the US, the annual incidence rates among children under 15 are 0.89 per million for AT/RT, 0.19 per million for renal MRTs, and 0.32 per million for extrarenal MRTs^81^). We addressed this limitation in part by validating our observations in a larger MRT cohort profiled using bulk sequencing, seeking to enhance the robustness and generalizability of our observations. Secondly, our study relied predominantly on bioinformatic analysis, and further experimental studies are necessary to confirm the functional implications of these observations. Even so, our results provide insights into the complex factors at play in shaping the MRT TIME and serve as a foundation for future studies.

## Supplementary Material

Supplementary Files

This is a list of supplementary files associated with this preprint. Click to download.

- SupplementaryTables.pdf
- SupplementaryFigures.docx
